# Molecular Epidemiology of Tuberculosis in a Sentinel Surveillance Population

**DOI:** 10.3201/eid0811.020403

**Published:** 2002-11

**Authors:** Barbara A. Ellis, Jack T. Crawford, Christopher R. Braden, Scott J. N. McNabb, Marisa Moore, Steve Kammerer, the National Tuberculosis Genotyping and Surveillance Network Work Group

**Affiliations:** * Centers for Disease Control and Prevention, Atlanta, Georgia, USA

**Keywords:** Sentinel surveillance, restriction fragment-length polymorphism, insertion sequence elements, risk factors, *Mycobacterium tuberculosis*

## Abstract

We conducted a population-based study to assess demographic and risk-factor correlates for the most frequently occurring *Mycobacterium tuberculosis* genotypes from tuberculosis (TB) patients. The study included all incident, culture-positive TB patients from seven sentinel surveillance sites in the United States from 1996 to 2000. *M. tuberculosis* isolates were genotyped by IS*6110*-based restriction fragment length polymorphism and spoligotyping. Genotyping was available for 90% of 11,923 TB patients. Overall, 48% of cases had isolates that matched those from another patient, including 64% of U.S.-born and 35% of foreign-born patients. By logistic regression analysis, risk factors for clustering of genotypes were being male, U.S.-born, black, homeless, and infected with HIV; having pulmonary disease with cavitations on chest radiograph and a sputum smear with acid-fast bacilli; and excessive drug or alcohol use. Molecular characterization of TB isolates permitted risk correlates for clusters and specific genotypes to be described and provided information regarding cluster dynamics over time.

**Figure:**
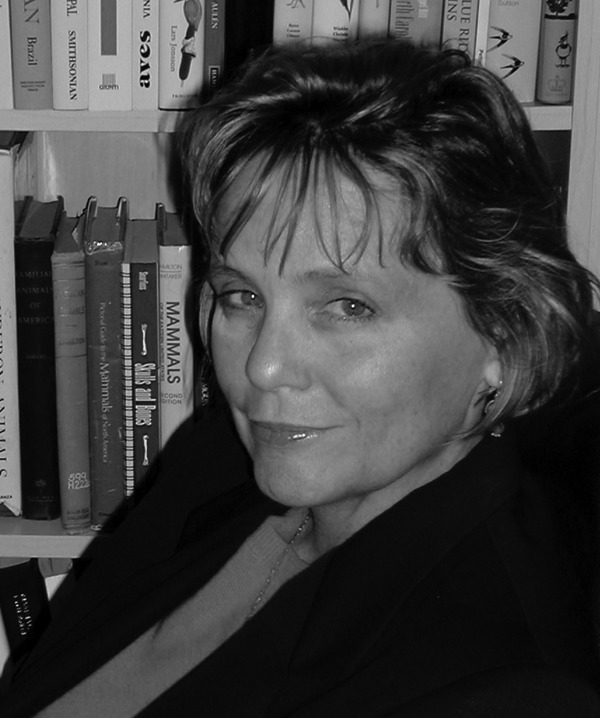
**Barbara A. Ellis, Guest Editor.** Dr. Ellis is a senior microbiologist with the National Center for Infectious Diseases, Centers for Disease Control and Prevention. Her research interests focus on the molecular epidemiology of infectious diseases, rodent-borne zoonotic diseases, and bioterrorism preparedness. Her work has included disease ecology studies of rodent-borne hemorrhagic fever viruses, molecular characterization of novel bartonellae, and molecular epidemiologic studies of Mycobacterium tuberculosis.

 Since 1990, characterization of *Mycobacterium tuberculosis* isolates by molecular methods has been useful in confirming suspected laboratory contamination and as an adjunct to epidemiology-based contact investigation (1–3). Most studies used the restriction fragment length polymorphism (RFLP) technique, s based on IS*6110* and specific to the *M. tuberculosis* complex. This genetic element may be present in different positions on the chromosome, resulting in a unique genotype useful for characterizing the strain of *M. tuberculosis* infecting a patient. Although RFLP has disadvantages (e.g., cost, time required to culture the organism, and specialized training and laboratory equipment), IS*6110*-based RFLP is the established method considered most discriminatory for genetic characterization of *M. tuberculosis* strains worldwide (4).

 In 1996, the Centers for Disease Control and Prevention (CDC) established seven sentinel surveillance sites in the United States (National Tuberculosis Genotyping and Surveillance Network) to assess the utility of molecular genotyping for improving tuberculosis (TB) prevention and control. The TB genotyping network used standardized protocols for molecular characterization of *M. tuberculosis* isolates from patients in all sentinel sites. The network was designed to address specific epidemiologic questions regarding the natural history, transmission, and potential applicability of molecular genotyping of *M. tuberculosis* strains to augment TB control activities (5). Two objectives were to identify and determine the prevalence of specific *M. tuberculosis* genotype clustering in populations of sentinel surveillance TB patients and to describe the demographic characteristics of these populations and the genotypic characteristics of *M. tuberculosis* strains in clustered and nonclustered TB cases. We describe demographic and risk factor correlates for the most frequently occurring *M. tuberculosis* genotypes in isolates collected from sentinel TB patients.

## Methods

This population-based sentinel study included all incident culture-positive TB patients from sentinel sites from January 1996 to December 2000. In brief, the seven sentinel surveillance sites included the states of Arkansas, Maryland, Massachusetts, Michigan, and New Jersey; Dallas, Tarrant, Cameron, and Hidalgo Counties in Texas; and Alameda, Contra Costa, Marin, San Mateo, Santa Clara, and Solano Counties in California. A detailed description of the study’s design, participants, population, and laboratory and epidemiologic methods is provided elsewhere (6).

All patients included in the study were reported to the CDC national TB case registry on the form Report of a Verified Case of Tuberculosis, a standardized electronic form submitted for TB surveillance to CDC by all state public health reporting areas. Data reported include patient demographics, laboratory test results, drug susceptibilities, information on chest radiographs, and treatment outcomes (7).

Investigators from the sentinel surveillance sites submitted patient isolates to the corresponding regional laboratory for genotyping and conducted routine contact investigations. In addition, participants from the surveillance sites performed detailed epidemiologic investigations on groups of persons with *M. tuberculosis* isolates that had matching genetic patterns or clusters (see below). The regional genotyping laboratories conducted IS*6110* RFLP on isolates from sentinel patients. Since low-copy numbers of IS*6110* (i.e., six or fewer copies) reduce test specificity, spacer oligonucleotide typing (spoligotyping) was conducted on such isolates. A cluster, which was identified by analysis of the entire TB genotyping network database, was defined as two or more isolates with either identical RFLP patterns (at least seven copies of IS*6110*) or identical RFLP and spoligotype patterns for isolates with RFLP patterns that had six or fewer copies of IS*6110*.

 Differences in the proportion of TB patients from the TB genotyping network population living in cities with populations of <100,000, 100,001 to 250,000, 250,001 to 500,000, and >500,000 were evaluated compared with those of the national TB patients for the year 2000 only. Statistics were obtained from the U.S. Census Bureau (available at: URL: http://www.census.gov/population/cen2000/phc-t6/tab04.pdf ).

Correlation of average TB incidence among cases at the seven sentinel sites and percentage of cases with isolates that clustered genetically were examined by year by using the Spearman rank correlation statistic. Clustering was determined by examining each year’s cases independently. A Mantel-Haenszel chi-square or Fisher exact test was used, as appropriate, to ascertain whether the sentinel population was representative of TB patients in the United States in terms of demographic, clinical, behavioral, or outcome characteristics.

 We used multiple logistic regression to assess the importance of demographic, clinical, behavioral, or outcome variables in predicting the occurrence of a given genotype for those genetic clusters that occurred most frequently (≥20 isolates). The dependent variable was the presence or absence of a given genotype. The best-fit logistic regression model was determined by the strategy of Hosmer and Lemeshow (8). In brief, a univariate analysis of the categorical independent variables was done by using the Mantel-Haenszel chi-square or Fisher exact test, as appropriate; any variable with a significance value of ≤0.20 was included in a best subset, multivariate logistic regression model. Collinearity of independent variables was assessed by using the variance/covariance matrix from PROC LOGISTIC (SAS Institute, Inc., Cary, NC) generate condition indices and a matrix of variance decomposition proportions to detect dependencies among the variables (9). Backward elimination of independent variables was performed if the probability of the independent variable was ≤0.20. Both the Wald statistic and 95% confidence interval were used on each coefficient to assess the significance of variables in each model; the log-likelihood ratio was used to assess the overall significance of the final models, and the Hosmer-Lemeshow statistic was used to evaluate the fit of each of the final models. Data were analyzed by SAS version 8.0 software (SAS Institute, Inc.) (10).

## Results

### Sentinel Population Characteristics

 The incidence of TB cases in the sentinel surveillance sites varied within and among sites over time (Table 1). From 1996 to 2000, the overall incidence of TB in the United States declined from 8.0 to 5.8 per 100,000 inhabitants, and similar downward trends were observed in each of the TB genotyping network sites. The California, New Jersey, Arkansas, and Texas sites had a higher incidence of TB than the overall national rates. The incidence rates in California and Texas (sites that included only six and four counties from each state) were similar to the overall incidence rates for each state (data not shown).

**Table 1:** Incidence of tuberculosis cases in the United States and in the sentinel surveillance areas of the National Tuberculosis Genotyping Surveillance Network, 1996–2000^a^

Sentinel surveillance site	1996	1997	1998	1999	2000	Mean
Arkansas	9.0	7.9	6.7	7.1	7.4	7.6
California^b^	16.3	13.9	13.9	12.9	11.6	13.7
Maryland	6.3	6.7	6.3	5.7	5.3	6.1
Massachusetts	4.3	4.4	4.6	4.4	4.5	4.4
Michigan	4.6	3.8	3.9	3.6	2.9	3.8
New Jersey	10.3	8.9	7.9	7.0	6.7	8.2
Texas^b^	12.7	12.8	12.5	10.9	9.6	11.7
United States	8.0	7.4	6.8	6.4	5.8	6.9

^a^Number per 100,000 inhabitants. ^b^Sentinel surveillance areas for California and Texas did not include the entire states.

In the surveillance area, 15,035 patients with verified TB represented 16% of the TB patients in the United States during the 5-year study period (Table 2). Overall, 11,923 TB patients were culture-positive (721 from Arkansas, 2,842 from California, 1,192 from Maryland, 1,022 from Massachusetts, 1,481 from Michigan, 2,599 from New Jersey, and 2,066 from Texas). Of TB patients in the surveillance areas, 79.3% (11,923) were culture positive, and RFLP results were available for 91.2% (10,883). However, spoligotyping results were not available for 131 of the isolates that had six or fewer copies of IS*6110* (5%; n=2,638); thus, these patients were excluded from our analysis. Of 1,171 isolates not genotyped by RFLP or spoligotyping, 12 (1%) were from Michigan, 35 (3%) from Maryland, 40 (3%) from Massachusetts, 110 (9%) from Arkansas, 156 (13%) from Texas, 327 (28%) from California, and 491 (42%) from New Jersey. Primary reasons for lack of genotyping results included inability to obtain cultures from private health-care providers, contamination of cultures, or poorly growing or nonviable cultures.

**Table 2:** Demographic and risk behavior factors and clinical, laboratory, and treatment outcomes for the sentinel surveillance patients (National Tuberculosis Genotyping Surveillance Network, compared with factors and outcomes of all tuberculosis patients, United States, 1996–2000^a,b^

Variable	Category	All U.S. TB cases (n=3,097) (%)	All NTGSN cases (n=15,035) (%)	Probability^c^
Gender	Male	58,356 (62.7)	8,767 (58.3)	<0.001
	Female	34,734 (37.3)	6,266 (41.7)	
	Unknown	7 (0.0)	2 (0.0)	
Age (yrs)	<4	3,289 (3.5)	518 (3.4)	NS
	5–14	2,397 (2.6)	393 (2.6)	NS
	15–24	7,988 (8.6)	1,462 (9.7)	<0.001
	25–44	32,433 (34.8)	5,413 (36.0)	0.005
	45–64	25,319 (27.2)	3,850 (25.6)	<0.001
	>64	21,662 (23.3)	3,397 (22.6)	NS
	Unknown	9 (0.0)	2 (0.0)	
Race/ethnicity	White, non-Hispanic	22,655 (24.3)	3,087 (20.5)	<0.001
	Black, non-Hispanic	30,201 (32.4)	4,775 (31.8)	NS
	Hispanic	20,475 (22.0)	2,923 (19.4)	<0.001
	American Indian/Native	1,280 (1.4)	38 (0.3)	<0.001
	Asian/Pacific Islander	18,346 (19.7)	4,195 (27.9)	<0.001
	Unknown	140 (0.2)	17 (0.1)	
Place of birth	U.S.-born	54,341 (58.4)	7,530 (50.1)	<0.001
	Foreign-born	38,252 (41.1)	7,468 (49.7)	
	Unknown	504 (0.5)	37 (0.2)	
Years in United States (foreign-born only)	<1	7,425 (19.4)	1,494 (20.0)	NS
	1	2,612 (6.8)	567 (7.6)	NS
	2	2,073 (5.4)	477 (6.4)	<0.005
	3	1,827 (4.8)	406 (5.4)	<0.05
	4	1,676 (4.4)	361 (4.8)	NS
	>5	19,396 (50.7)	3,688 (49.4)	<0.001
	Unknown	3,243 (8.5)	475 (6.4)	
Country of origin^d^	Philippines	4,862 (12.7)	1,113 (14.9)	<0.0001
	Mexico	8,795 (23.0)	1,100 (14.7)	<0.0001
	Vietnam	3,824 (10.0)	968 (13.0)	<0.0001
	India	2,527 (6.6)	883 (11.8)	<0.0001
	China	1,930 (5.0)	370 (5.0)	NS
	Haiti	1,470 (3.8)	225 (3.0)	<0.0005
	Peru	636 (1.7)	207 (2.8)	<0.0001
	Republic of Korea	1,176 (3.1)	202 (2.7)	NS
	Ethiopia	578 (1.5)	153 (2.0)	<0.001
	Ecuador	627 (1.6)	115 (1.5)	NS
	Other	11,827 (30.9)	2,132 (28.5)	<0.0001
Status at diagnosis	Alive	90,141 (96.8)	14,611 (97.2)	0.02
	Dead	2,925 (3.1)(	422 (2.8)	
	Unknown	31 (0.0)	2 (0.0)	
Site of disease	Pulmonary	68,611 (73.7)	10,576 (70.3)	<0.001
	Extrapulmonary	17,406 (18.7)	3,210 (21.4)	<0.001
	Pulmonary and Extrapulmonary	7,046 (7.6)	1,241 (8.3)	0.003
	Unknown	34 (0.0)	8 (0.1)	
Primary disease site	Pulmonary	73,157 (78.6)	11,365 (75.6)	<0.0001
	Lymph: cervical	4,312 (4.6)	1,020 (6.8)	<0.0001
	Pleural	3,842 (4.1)	674 (4.5)	<0.05
	Miliary	1,407 (1.5)	241 (1.6)	NS
	All other	10,345 (11.1)	1,727 (11.5)	NS
	Unknown	34 (0.0)	8 (0.0)	
Sputum smear for acid-fast organisms	Negative	36,912 (39.6)	5,995 (39.9)	<0.0001
	Positive	33,235 (35.7)	4,735 (31.5)	
	Not done/unknown	22,950 (24.6)	4,305 (28.7)	
TST at diagnosis	Negative	13,215 (14.2)	1,947 (12.9)	<0.001
	Positive	54,113 (58.1)	8,799 (58.5)	
	Not done/unknown	25,769 (27.6)	4,289 (28.6)	
Case verification criteria	Positive culture	74,940 (80.5)	11,967 (79.6)	<0.01
	Positive smear	765 (0.8)	136 (0.9)	NS
	Clinical case	11,286 (12.1)	1,858 (12.4)	NS
	Provider diagnosis	6,106 (6.6)	1,074 (7.1)	<0.01
Chest radiograph^d^	Cavitary	18,742 (24.8)	2,990 (25.3)	NS
	Noncavitary	50,652 (66.9)	7,897 (66.8)	NS
	Normal	2,495 (3.3)	360 (3.0)	NS
	Not done/unknown	3,802 (5.0)	578 (4.9)	
	Total	75,691	11,825	
HIV status^e^	Positive	6,062 (18.8)	884 (16.7)	NS
	Negative	16,525 (51.2)	2,406 (45.5)	
	Indeterminate	47 (0.1)	6 (0.1)	
	Refused	1,959 (6.1)	325 (6.1)	
	Not offered	4,130 (12.8)	899 (17.0)	
	Test done, unknown	714 (2.2)	115 (2.2)	
	Unknown	2,812 (8.7)	658 (12.4)	
	Total	32,249	5,293	
Homeless within past year	Yes	5,789 (6.2)	646 (4.3)	<0.001
	No	84,873 (91.2)	14,185 (94.3)	
	Unknown	2,435 (2.6)	204 (1.4)	
Resident of correctional facility at diagnosis	Yes	3,352 (3.6)	377 (2.5)	<0.001
	No	89,479 (96.1)	14,617 (97.2)	
	Unknown	266 (0.3)	41 (0.3)	
Correctional facility type	Federal prison	164 (4.9)	6 (1.6)	<0.005
	State prison	1,036 (30.9)	97 (25.7)	<0.05
	Local jail	1,905 (56.8)	231 (61.3)	NS
	Juvenile facility	33 (1.0)	8 (2.1)	NS
	Other	161 (4.8)	34 (9.0)	<0.001
	Unknown	53 (1.6)	1 (0.3)	
	Total	3,352	377	
Resident, long-term care facility at diagnosis	Yes	3,157 (3.4)	441 (2.9)	0.004
	No	89,656 (96.3)	14,552 (96.8)	
	Unknown	284 (0.3)	42 (0.3)	
Long-term care facility type	Nursing home	1,794 (56.8)	279 (63.3)	<0.01
	Hospital-based	441 (14.0)	66 (15.0)	NS
	Residential	356 (11.3)	34 (7.7)	<0.05
	All other	504 (16.0)	55 (12.5)	NS
	Unknown	62 (2.0)	7 (1.6)	
	Total	3,157	441	
Injecting drug use^f^	Yes	2,569 (2.8)	515 (3.4)	<0.001
	No	83,141 (89.3)	13,771 (91.6)	
	Unknown	7,387 (7.9)	749 (5.0)	
Noninjecting drug use^f^	Yes	6,557 (7.0)	811 (5.4)	<0.001
	No	78,622 (84.5)	13,367 (88.9)	
	Unknown	7,918 (8.5)	857 (5.7)	
Excessive alcohol use^g^	Yes	13,646 (14.7)	1,661 (11.0)	<0.001
	No	71,924 (77.3)	12,552 (83.5)	
	Unknown	7,527 (8.1)	822 (5.5)	
Drug resistance^h^				
First-line drugs	Yes	8,456 (11.7)	1,482 (12.6)	<0.001
	No	57,029 (79.0)	8,886 (75.5)	
	Not tested/unknown	6,703 (9.3)	1,399 (11.9)	
	Total	72,188	11,767	
Second-line drugs	Yes	1,341 (1.9)	208 (1.8)	<0.001
	No	175 (0.2)	78 (0.7)	
	Not tested/unknown	70,672 (97.9)	11,481 (97.6)	
	Total	72,188	11,767	
DOT	Yes—total DOT	40,511 (43.5)	4,936 (32.8)	<0.001
	Yes—both DOT and self-administered	20,555 (22.1)	3,648 (24.3)	<0.001
	No	23,337 (25.1)	5,326 (35.4)	<0.001
	Unknown	8,694 (9.3)	1,125 (7.5)	
Within city limits	Yes	80,775 (86.8)	14,603 (97.1)	<0.001
	No	10,916 (11.7)	374 (2.5)	
	Unknown	1,406 (1.5)	58 (0.4)	
Previous diagnosis of TB	Yes	4,794 (5.1)	652 (4.3)	<0.001
	No	87,567 (94.1)	14,336 (95.4)	
	Unknown	736 (0.8)	47 (0.3	
Duration of therapy (days)	Mean	246	245	NS
	Median	217	214	
	Std. dev.	135	130	
	No.	65,344	10,822	

^a^NTGSN, National Tuberculosis Genotyping Surveillance Network;TB, tuberculosis; DOT, directly observed therapy; TST, tuberculin skin test; Std. dev., standard deviation; NS, not significant (p>0.05). .^b^Subtotals for each category are listed if different from the total case numbers. ^c^Probability of significant differences between U.S. TB patients and all NTGSN surveillance patients (chi-square test; *t*-test for duration of therapy); referent group is all other groups combined, excluding not done or unknown categories, unless otherwise noted. ^d^Top 10 countries for foreign-born patients only. ^e^Excludes cases with extrapulmonary TB only. ^f^HIV cases from California are excluded because this site does not report HIV results on Report of a Verified Case of Tuberculosis forms; ages 15–44 years only. ^g^Injecting or noninjecting drug use within last year; includes use of licensed, prescription, or illegal drugs (not prescribed by a physician). ^h^Excessive use of alcohol within the past year as indicated by participation in alcohol treatment programs, diagnosis of alcoholism, or observation of intoxication during visits to health-care facilities. ^i^Drug resistance on initial testing of isolate. First-line drug resistance is resistance to at least one of the following: isoniazid, rifampin, ethambutol, or streptomycin. Second-line drug resistance is resistance to one or more of the following: ethionamide, kanamycin, cycloserine, capreomycin, para-amino salicylic acid, amikacin, rifabutin, ciprofloxacin, ofloxacin, or other drugs. Testing results for one or more of the drugs could have been missing.

Characteristics of the TB patient population from the genotyping network sentinel sites were comparable with those from the entire United States, with some exceptions (Table 2). Sentinel surveillance populations had higher proportions of women (42% for the genotyping network vs. 37% for the United States overall) and patients in the 15- to 44-year age category, and were more often homeless or lived in correctional or long-term care facilities. Higher proportions of genotyping network patients used intravenous drugs, but fewer patients used noninjecting drugs or alcohol excessively.

 Of the study population, about 4% reported previous episodes of TB (652 of 15,035; Table 2). Of persons with a previous recent history of TB, 28 had TB after completing >1 year of therapy within the study period; genotyping data on isolates from both episodes were available for 22 of these persons. A higher number of persons from the TB genotyping network study population lived within city limits (97% vs. 87%). However, when compared with national averages, genotyping network populations were generally from smaller towns and cities: 1,446 (69%) of 2,099 genotyping network patients were from cities and towns with <250,000 inhabitants, compared with 10,093 (62%) of 16,377 TB patients nationwide (Mantel-Haenszel chi square= 41.8; p<0.0001).

 The proportion of foreign-born patients was higher in genotyping network populations compared with the overall national average (50% for genotyping network vs. 41% for the United States). Numbers of foreign-born TB patients increased over time at about the same rate for both genotyping network populations and national TB patients. From 1996 to 2000, national proportions of foreign-born TB patients increased from 37% (7,725/21,045) to 47% (7,593/16,281); in the genotyping network populations, the proportions of foreign-born TB patients increased from 44% (1,153/2,642) to 58% (1,222/2,092). Characteristics of the genotyping network population between sites were similar, as were culture-positive genotyping network populations compared with the overall genotyping network case population.

### Analysis of Genotyping Data

 The distribution and diversity of RFLP and spoligotyping pattern results from the genotyping network have been discussed in detail (11). In contrast to that analysis, we used both RFLP and spoligotyping results to define genetic clusters. Overall, 6,609 distinct patterns were identified, including 1,029 that contained ≥2 isolates per cluster. When analyzed by site, 1,018 clusters were identified: 71 clusters were from Arkansas (611 cases genotyped, 2–16 cases per cluster), 233 from California (2,511 cases, 2–128 cases per cluster), 104 from Maryland (1,157 cases, 2–36 cases per cluster), 85 from Massachusetts (982 cases, 2–16 cases per cluster), 125 from Michigan (1,469 cases, 2–102 cases per cluster), 196 from New Jersey (2,112 cases, 2–40 cases per cluster), and 204 from Texas (1,910 cases, 2–96 cases per cluster). Overall, 970 distinct genotypes, including 235 representing clusters, had ≤6 copies (2,507 cases, 24% clustered, 2–93 cases per cluster). In contrast, 794 clusters from 5,639 distinct genotypes had ≥ 7 IS6110 copies (8,245 cases, 14% clustered, 2–105 cases per cluster). Most clusters included seven or fewer persons (85%; 900/1,029).

### Longitudinal Analysis

 Most clusters occurred in only a single site (66%; 680/1,029). However, 260 (25%) were found in two sites, 55 (5%) in three sites, 19 (2%) in four, 8 (1%) in five, and 7 (1%) in six sites. As expected, clusters that spanned multiple sites were larger. Clusters found at a single site averaged four persons per cluster (mean=3.65; standard error [SE] ± 0.22; n=680), in contrast to 61 persons per cluster for the genotypes found at six sites (mean=61.14; SE ± 23.6; n=7; Kruskal-Wallis test, p<0.0001). Most (62%) of the 34 clusters that occurred in at least four sites occurred in all 5 years of the study; 26% in 4 years; and 6% each in 3 and 2 years of the study.

**Figure 1:**
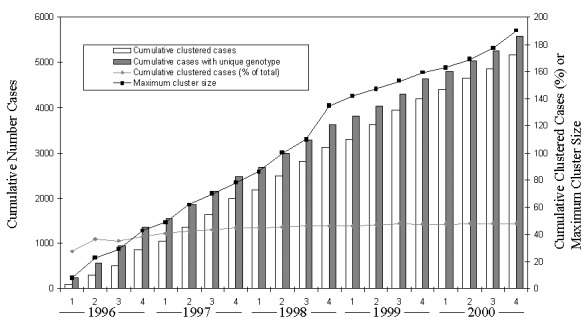
Numbers of tuberculosis cases, cumulative proportion of cases with isolates in genetic clusters, and maximum genetic cluster size from seven sentinel surveillance sites by quarter that verified case was counted, 1996–2000. Numbers of cases with isolates that had unique genotypes and those with isolates that were in genetic clusters are shown separately.

 Changes in proportions of patients with isolates that clustered were observed over time. In the first 2 years of the study, the percentage of the cumulative total number of cases that clustered increased from 28% to 45%; smaller increases occurred thereafter (Figure 1). Overall, the proportion of clustered cases was 48% (5,171/10,752). The percentages of clustered cases by sites were 28% (276/982) for Massachusetts; 34% (393/1,157) for Maryland; 41% (873/2,112) for New Jersey; 42% (1,046/2,511) for California; 44% (266/611) for Arkansas; 49% (720/1,469) for Michigan; and 57% (1,093/1,910) for Texas. Maximum cluster size and absolute numbers of cases with isolates that clustered continued to increase through the end of the study.

**Figure 2:**
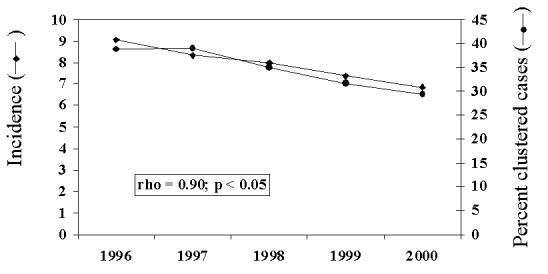
Average annual incidence of tuberculosis for seven sentinel surveillance sites and percentage of cases with isolates in genetic clusters, 1996 to 2000. Spearman correlation coefficient and probability of correlation between incidence and percentage of cases clustered are given.

 Overall, cases with isolates that clustered showed a concomitant decline with average incidence of TB over the 5-year period (Figure 2). A significant positive association was observed between the percentage of cases with clustered genotypes and TB incidence over time (Spearman rho=0.90; p=0.037).

### Risk Factor Analyses of Genetic Clusters

 Compared with persons whose isolates had unique genotypes, persons with isolates that clustered were more likely to be non-Hispanic, black men born in the United States. They were more likely to have pulmonary disease and abnormal chest radiographs with cavities; in addition, they more often had positive sputum smears; were HIV-positive, homeless, or residents of a correctional facility; and used drugs or alcohol excessively (Table 3). Patients with unclustered isolates were 5 years older on the average than those with isolates that clustered (44.8 years vs. 49.4 years, respectively; Table 3). Multiple logistic regression efforts resulted in models that were not robust (data not shown).

**Table 3:** Comparison of demographic and behavioral risk factors and clinical and treatment outcomes of tuberculosis (TB) case-patients who have genetically clustered genotypes with factors and outcomes of patients who had unique genotype patterns^a^

Variable^b^		Clustered (%)		Unclustered (%)	Relative risk (95% CI)	Probability^c^
Total cases (n=10,752)		5.171(48.1)		5,581 (51.9)		
Gender	Male	3,289 (63.6)		3,107 (55.7)	1.19 (1.14% to 1.24%)	<0.001
	Female	1,881 (36.4)		2,473 (44.3)		
	Unknown	1 (0.0)		1 (0.0)		
Mean age (yrs; ±S.E.)		44.8 (±0.26)		49.4 (±0.28)		<0.0001
Race/ethnicity	White, non-Hispanic	1,018 (19.7)		1,201 (21.5)	0.94 (0.90% to 0.99%)	0.02
	Black, non-Hispanic	2,254 (43.6)		1,237 (22.2)	1.61 (1.55% to 1.67%)	<0.001
	Hispanic	914 (17.7)		1,112 (19.9)	0.92 (0.88% to 0.97%)	0.003
	American Indian/Native	17 (0.3)		10 (0.2)		
	Asian/Pacific Islander	961 (18.6)		2,014 (36.1)	0.60 (0.56% to 0.63%)	<0.001
	Unknown	7 (0.1)		7 (0.1)	0.59 (0.55% to 0.63%)	
Place of birth	U.S.-born	3,331 (64.4)		2,023 (36.2)	1.83 (1.75% to 1.90%)	<0.001
	Foreign-born	1,825 (35.3)		3,552 (63.6)		
	Unknown	15 (0.3)		6 (0.1)		
Recent to United States^d^	Yes	535 (29.3)		1,225 (34.5)	2,111 (59.4)	<0.001
	No	1,181 (64.7)				
	Unknown	109 (6.0)		216 (6.1)		
Site of disease	Pulmonary	3,902 (75.5)		3,835 (68.7)	1.20 (1.14% to1.26%)	<0.001
	Extrapulmonary	788 (15.2)		1,254 (22.5)	0.77 (0.72% to 0.81%)	<0.001
	Pulmonary and extrapulmonary	476 (9.2)		492 (8.8)		NS
	Unknown	5 (0.1)		0		
Sputum smear	Positive	2,270 (43.9)		2,011 (36.0)	1.22 (1.11% to 1.33%)	<0.001
	Negative	1,802 (34.8)		1,943 (34.8)		
	Not done/unknown	1,099 (21.3)		1,627 (29.1)		
Chest radiograph^e^	Cavitary	1,345 (30.7)		1,172 (27.1)	1.09 (1.04% to 1.14%)	<0.001
	Noncavitary	2,639 (60.2)		2,826 (65.3)		
	Normal	146 (3.3)		118 (2.73)		
	Not done/unknown	253 (5.8)		211 (4.9)		
	Total	4,383		4,327		
HIV status^f^	Positive	458 (22.2)		223 (11.8)	1.37 (1.29% to 1.46%)	<0.001
	Negative	978 (47.4)		847 (44.8)		NS
	Indeterminate	0		4 (0.2)		
	Refused	106 (5.1)		138 (7.3)		
	Not offered	252 (12.2)		354 (18.7)		
	Unknown	270 (13.0)		323 (17.1)		
	Total	2,064		1,889		
Homeless within past year	Yes	370 (7.2)		139 (2.5)	1.55 (1.46% to 1.64%	<0.001
	No	4,724 (91.4)		5,370 (96.2)		
	Unknown	77 (1.5)		72 (1.3)		
Resident of correctional facility at diagnosis	Yes	190 (3.7)		69 (1.2)	1.55 (1.43% to 1.67%)	<0.001
	No	4,966 (96.0)		5,503 (98.6)		
	Unknown	15 (0.3)		9 (0.2)		
Injecting drug use^g^	Yes	312 (6.0)		72 (1.3)	1.73 (1.65% to 1.83%)	<0.001
	No	4,540 (87.8)		5,231 (93.7)		
	Unknown	319 (6.2)		278 (5.0)		
Noninjecting drug use^g^	Yes	460 (8.9)		140 (2.5)	1.65 (1.57% to 1.73%)	<0.001
	No	4,335 (83.8)		5,140 (92.1)		
	Unknown	376 (7.3)		301 (5.4)		
Excessive alcohol use^g^	Yes	948 (18.3)		371 (6.6)	1.61 (1.54% to 1.67%)	<0.001
	No	3,897 (75.4)		4,893 (87.7)		
	Unknown	326 (6.3)		317 (5.7)		
First-line drugs ^h^						
	Yes	622 (12.1)		755 (13.7)	0.93 (0.87% to 0.99%)	0.016
	No	2,718 (53.0)		3,337 (60.5)		
	Not done	1,748 (34.1)		1,356 (24.6)		
	Unknown	45 (0.9		66 (1.2)		
	Total	5,133		5,514		

^a^CI, confidence interval; S.E., standard error. ^b^Only factors that had significant differences are shown. ^c^Probability of chi-square statistic is shown, except for t-test results from analysis of age from each group. ^d^Foreign-born only; arrived in the United States within 2 years. ^e^Excludes cases with extrapulmonary TB only. ^f^California TB cases not included; ages 15–44 years only. ^g^Excessive drug or alcohol use within last year. ^h^First-line drug resistance is resistance to at least one of the following: isoniazid, rifampin, ethambutol, or streptomycin. Second-line drug resistance is resistance to one or more of the following: ethionamide, kanamycin, cycloserine, capreomycin, para-amino salicylic acid, amikacin, rifabutin, ciprofloxacin, ofloxacin, or other drugs. Testing results for one or more of the drugs could have been missing.

**Figure 3:**
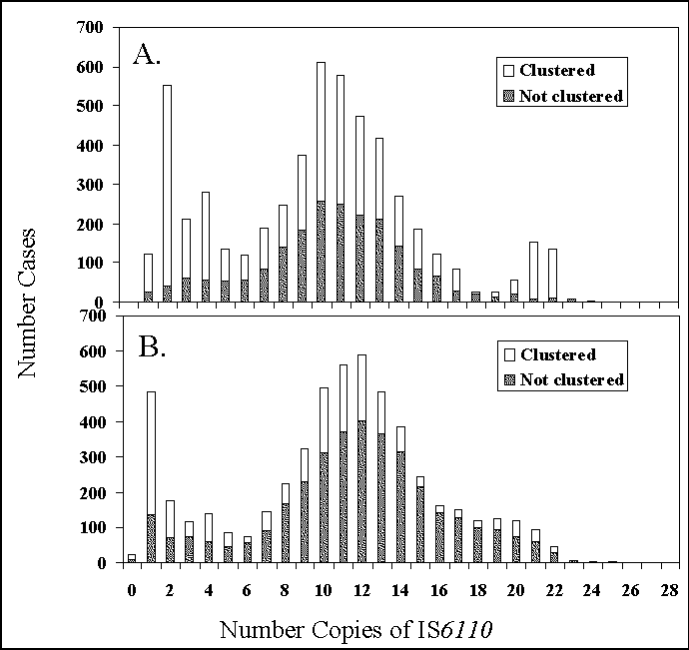
Number of cases with isolates that had unique genotypes (“not clustered”) and those in genetic clusters for U.S.-born (A) and foreign-born persons (B) by number of copies of IS*6110*.

 Except for 4 genotypes, all 34 clusters with ≥ 20 isolates per cluster had significant demographic, clinical, and behavioral risk factors (Table 4). Race, ethnicity, and place of birth were frequently significant predictors for a given genotype. Other predictors included gender, age, site of disease, resistance to first-line drugs, and alcohol or drug abuse (Table 4). Twelve (40%) of 30 of these larger clusters were observed in four or more sites over a 5-year period. Lower percentages of foreign-born patients than U.S.-born patients clustered, regardless of the number of IS*6110* copies (Figure 3). More than 50% (1,025/1,825) of the foreign-born patients whose isolates clustered had been in the United States for ≥5 years. Clustering of isolates from foreign-born patients ranged from 15% (49/316) in Michigan to 38% (309/816) in Texas.

**Table 4:** Odds ratios from best-fit logistic regression analyses of the presence or absence of a specific genetic cluster of *Mycobacterium tuberculosis* on demographic, clinical, behavioral, or treatment outcome variables ^a^

Designation^c^	IS*6110* copies	Spoligotype^c^	N	Main effect	Odds ratio estimates (95% CI)^b^	Wald p^b^
00003^c^	1	777777777760771	40	Asian/Pacific Islander	3.70 (1.51% to 9.02%)	0.004
				Age	0.98 (0.96% to 0.99%)	0.017
				Foreign-born	12.4 (3.83% to 39.9%)	<0.0001
00129^d^	1	777777777413771	25	Asian/Pacific Islander	73.3 (17.0% to 315.6%)	<0.0001
				Extrapulmonary infection	2.57 (1.10% to 6.03%)	0.03
00129^d^	1	777777774413771	83	Asian/Pacific Islander	282.8 (88.06% to 908.11%)	<0.0001
00129^d^	1	477777777413071	23	Asian/Pacific Islander	6.34 (1.52% to 26.44%)	0.01
				Foreign-born	10.4 (1.55% to 70.12%)	0.02
00129^d^	1	777777777413731	13	Asian/Pacific Islander	13.88 (3.71% to 51.92%)	<0.0001
				Resistance to first-line drugs^d^	3.80 (1.22% to 11.86%)	0.02
00129	1	777776407760601	40	Female	2.73 (1.43% to 5.23%)	0.0025
				Black, non-Hispanic	3.57 (1.47% to 8.68%)	0.005
				Injecting drug use	3.81 (1.81% to 8.03%)	0.0004
00016	2	701776777760601	129	Male	0.58 (0.40% to 0.84%)	0.004
				Black, non-Hispanic	10.88 (5.48% to 21.6%)	0.006
00016^c^	2	777776777760771	82	Hispanic	16.36 (10.15% to 26.37%)	<0.0001
00016	2	037776777760601	30	Age	1.03 (1.01% to 1.05%)	0.006
				Black, non-Hispanic	7.13 (2.36% to 21.53%)	0.0005
				Resident, long-term care facility	3.67 (1.17% to 11.70%)	0.026
00016^d^	2	777776777760601	175	U.S.-born	3.12 (1.85% to 5.26%)	<0.0001
				Excessive alcohol use	0.55 (0.37% to 0.83%)	0.0048
00370	3	700036777760731	13	White, non-Hispanic	5.20 (1.52% to 17.79%)	0.0087
				HIV positive	5.87 (1.69% to 20.41%)	0.005
				Noninjecting drug use	3.74 (1.17% to 12.01%)	0.03
00017^d^	4	700076777760771	25	Hispanic	4.97 (2.16% to 11.44%)	0.0002
00017^d^	4	777776777760771	64	Hispanic	15.7 (9.24% to 26.71%)	<0.0001
01285	4	777776777760771	20	Resident, correctional facility	8.23 (3.08% to 22.01%)	<0.0001
00015	7		28	Black, non-Hispanic	7.04 (1.64% to 30.3%)	0.0087
				Injecting drug use	4.84 (2.11% to 11.09%)	0.0002
				Excessive alcohol use	2.28 (1.02% to 5.13%)	0.05
00768	9		19	Black, non-Hispanic	11.68 (1.54% to 88.87%)	0.02
				Noninjecting drug use	2.77 (1.11% to 6.92%)	0.03
00242^d^	10		95	Male	2.12 (1.27% to 3.56%)	0.004
				Age	0.97 (0.96% to 0.98%)	<0.0001
				U.S.-born	8.44 (2.63% to 27.09%)	0.0003
				Homeless	3.60 (2.16% to 5.98%)	<0.0001
				Noninjecting drug use	0.46 (0.24% to 0.90%)	0.02
00028	11		70	Black, non-Hispanic	17.57 (5.50% to 56.12%)	<0.0001
00159	11		24	Excessive alcohol use	2.76 (1.23% to 6.22%)	0.01
00325	11		20	Age	1.03 (1.01% to 1.06%)	0.01
				Excessive alcohol use	3.08 (1.22% to 7.70%)	0.02
00673	11		25	Asian/Pacific Islander	84.6 (19.85 to 361.9%)	<0.0001
00757	11		16	Age	0.90 (0.85% to 0.94%)	<0.0001
				HIV positive	4.86 (1.60% to 14.79%)	0.005
00019^c^	12		27	Male	3.68 (1.10% to 12.39%)	0.03
				White, non-Hispanic	5.4 (2.35% to 11.08%)	<0.0001
00372	12		20	Homeless	6.09 (2.43% to 15.20%)	0.0001
				Resident, long-term care facility	5.52 (1.535 to 20.0%)	0.009
00035	13		33	Black, non-Hispanic	6.96 (2.3% to 21.0%)	0.0006
				Resistance to second-line drugs^e^	40.59 (16.5% to 99.85%)	<0.0001
00867	14		20	Black, non-Hispanic	11.68 (1.54% to 88.87%)	0.02
				Noninjecting drug use	2.77 (1.11% to 6.92%)	0.03
01284	17		46	Black, non-Hispanic	2.40 (1.22% to 3.57%)	<0.0001
				Pulmonary disease	0.92 (-0.01% to 1.86%)	0.054
00237^c^	21		98	White, non-Hispanic	2.80 (1.81% to 4.33%)	<0.0001
				Excessive alcohol use	2.09 (1.36% to 3.22%)	0.0007
01693	21		29	HIV positive	3.16 (1.39% to 7.18%)	0.006
				Injecting drug use	3.08 (1.26% to 7.56%)	0.014
				Extrapulmonary disease	3.99 (1.69, 9.42)	0.002
00027	22		78	Black, non-Hispanic	1.74 (1.05% to 2.90%)	0.03
				Sputum-smear positive	3.07 (1.75% to 5.39%)	<0.0001

aCI, confidence interval. bOnly genetic clusters that had ≥20 isolates were included in the analysis; some samples sizes are <20 because of missing data among independent variables (Wald 95% confidence intervals given in parentheses). Only genetic clusters with significant predictors are listed. Age was modeled as a continuous variable. cThe National Tuberculosis Genotyping Surveillance Network (NTGSN) designation for the IS6110 RFLP pattern is represented; spoligotype octal code designations are presented only for those genetic clusters from isolates that had ≤6 copies of IS6110. RFLP patterns and spoligotypes are detailed elsewhere (11). dsolates observed in ≥ 4 sites over 5 years. e First-line drug resistance is resistance to at least one of the following: isoniazid, rifampin, ethambutol, or streptomycin. Second-line drug resistance is resistance to one or more of the following: ethionamide, kanamycin, cycloserine, capreomycin, para-amino salicylic acid, amikacin, rifabutin, ciprofloxacin, ofloxacin, or other drugs.

## Discussion

 This population-based study is the largest that has been conducted in the United States to assess risk factors related to specific M. tuberculosis genotypes. Generally, clustered isolates have been considered recently acquired infections (12). However, this assumption may not always be correct. Clustering does not prove that transmission occurred, and its demonstration depends on adequate sampling of the population, incidence of TB, and characteristics of the study population (e.g., age structure, population mobility, duration of residence, and immune status) (1,13) . Only 25%–42% of patients in genetic clusters were shown to have epidemiologic connections with another member of the cluster (14–16). Conventional epidemiologic investigation of these TB patients (including interviews) was conducted, but inclusion in this analysis was outside the scope of this article. Thus, results that indicate clustered genotypes are representative of recent transmission should be interpreted with caution.

 Given this caveat, our results nevertheless demonstrate several consistent patterns. Differences in demographic and other risk factors for persons with isolates that clustered corroborated those from smaller studies conducted in the United States and larger surveys in Europe. Extensive surveys from the Netherlands (17) also demonstrated that persons with isolates that clustered genetically were younger than those with unique genotypes. Other risk factors for clustering included being male, born in the United States, non-Hispanic black, or homeless; using drugs and alcohol excessively; and having pulmonary disease and cavitations on chest radiograph, a sputum smear with acid-fast bacilli, and HIV infection. These risk factors have been observed for TB patients in different communities (12,18,19). The heterogeneity and diversity of the study population may account for our failure to produce a multivariate logistic model to predict clustering.

 A third of the foreign-born cases were recent immigrants to the United States, and overall, the percentage of clustered isolates from foreign-born persons was lower than the percentage from nonimmigrants (Figure 3), indicating that at least a portion of these cases resulted from reactivation of latent disease or recent infection in the country of origin. In addition, for foreign-born persons, clustering of M. tuberculosis increased with the duration of residence in the United States. These results suggest that recently imported strains of M. tuberculosis from foreign-born persons may not commonly spread to U.S. residents or that transmission may be occurring after a lag time before the imported strains manifest as disease in contacts. Similar observations have been published in studies from San Francisco, New York, Switzerland, and Norway (20–24). These data may also reflect gaps in our knowledge of M. tuberculosis genotypes in circulation; a comparison of the U.S. TB genotyping network results with other databases worldwide may be warranted.

 Logistic regression analysis of the most commonly occurring strains demonstrated that different risk factors were associated with specific genotypes. Several genotypes were associated with ethnic origin (e.g., Asian or Pacific Islander and Hispanic patients with six and three genotypes, respectively; Table 4). A recent study in Norway showed that several clusters consisted of patients of the same ethnic origin (23). An association has also been observed between the patient’s ethnic origin and IS6110 copy number (25). These results, in conjunction with additional epidemiologic data, may be useful in tracking the geographic origin and spread of M. tuberculosis strains of public health importance (26).

 A small proportion of clustered isolates were from persons from more than four sites spanning 5 years of study (Table 4). Although an in-depth analysis of epidemiologic links was not possible in this study, we found no evidence of recent transmission between patients with identical genotypes from the different states (data not shown); this lack of transmission was also noted in a smaller study in the United States (27). Since TB transmission is generally considered a local event, these ubiquitous genotypes may be widespread because of social factors (e.g., homelessness or alcohol or drug abuse; Table 4). In addition, these genotypes may represent older, endemic domestic strains that have been in the United States for centuries and have dispersed more widely throughout the United States than the more recently imported strains. Further molecular characterization of these genotypes may show additional differences not detected by RFLP. Nonetheless, the effect of M. tuberculosis virulence or host factors on the distribution of these genotypes cannot be ascertained.

 The proportion of strains that were classified into clusters of identical genotypes (48%) was comparable with proportions in the Netherlands and Denmark (50%) (2,28), but the proportion was considerably higher than in two other countries (17% in Switzerland [29]; 20% in Norway [23]). The cumulative percentage of clustered strains reached a plateau by the end of the study’s second year (Figure 1), a finding consistent with other molecular epidemiologic TB studies (2). Increases in maximum cluster size were anticipated because, as sample sizes increase with time, the number of isolates in each cluster would be expected to increase. In addition, higher proportions of clustered cases were observed for low-band number patterns (Figure 3), which had the maximum cluster size and may indicate that the low-copy IS6110 patterns are not specific, even with the addition of spoligotyping.

 The sensitivity and specificity of IS6110 RFLP in molecular epidemiologic studies have not been quantified and represent a potential limitation of this study. Although the stability of IS6110 is relatively high, the half-life of IS6110 RFLP is estimated to be 3–10 years (29–31) based on typing of serial isolates from individual patients. A study of isolates from patients in confirmed chains of transmission showed little change in IS6110 patterns (32). Calculation of these rates may be influenced by the duration between time of disease onset and time of sampling and may be proportional to the effectiveness of the TB control program (30). Because genotyping results were not available for 10% of TB cases in this study, estimates of the degree of clustering and the size of clusters are conservative. Some unique isolates might have clustered if some of the missing isolates had been aaavailable or if other cases with the same strain were present outside the study area (33).

 Sentinel surveillance sites defined by artificial boundaries (i.e., state lines) not entirely representative of TB patients from the United States were included in this study. More than 90% of the isolates from patients from the surveillance areas were genotyped, and these isolates were representative of those culture-positive patients from the sentinel surveillance areas. However, 16% of all TB case-patients reported in the United States were included in these sentinel surveillance sites during the 5-year study period. In addition, the sentinel surveillance population had higher proportions of foreign-born persons than the national average. Because of the propensity of foreign-born persons to have isolates with unique genotypes, the actual rate of clustering may have been underestimated. Nonetheless, sentinel surveillance of TB cases has provided a useful method for documenting genotypes in circulation in the United States and for identifying risk factor correlates of common genotypes.

 Annual declines in TB incidence were paralleled by similar declines in the proportion of cases with genotypes in clusters (Figure 2), a finding consistent with the hypothesis that decreased clustering is expected with declining incidence (20). Since effort was similar each year, this association is not likely to be an artifact related to sample size (i.e., as sample size or number of cases becomes smaller, the probability of detecting clusters decreases). These findings underscore the importance of long-term longitudinal molecular studies and the potential usefulness of these methods in evaluating program effectiveness and improving program management.
